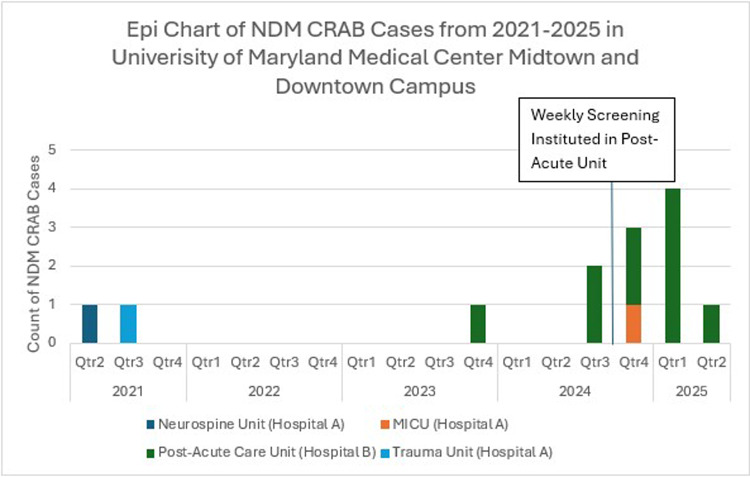# 241 Efficacy of 2 commercial glycol vapor products in reducing aerosolized bacteriophage MS2 and healthcare-associated pathogens on surface

**DOI:** 10.1017/ash.2026.10614

**Published:** 2026-06-23

**Authors:** Gaayathri Krishnan, Tracy Hazen, Ibrahim El-Imam, J. Kristie Johnson, Gwen Robinson, Sierra Spindler, Mike Humphrys, Surbhi Leekha, Japria Davis, Amber Thomas, Jinshui Fan, Calaija Phillips, Karinda Felton, Leigh Smith

**Affiliations:** 1 University of Maryland; 2 University of Maryland School of Medicine; 3 University of Maryland, Baltimore; 4 University of Maryland Baltimore; 5 University of Maryland Medical System; 6 University of Maryland Medical Center; 7 Maryland Department of Health

## Abstract

**Introduction:** NDM-producing Carbapenem-resistant Acinetobacter baumannii (CRAB) is an emerging concern globally due to extensive antimicrobial resistance and high mortality, although less common in the United States. We describe the epidemiology of this pathogen at our academic medical center over a 5-year period. **Methods:** Observational study and epidemiologic investigation at two campuses (800-bed hospital (“A”) including 7 adult ICUs and 144-bed hospital (“B”) including 1 ICU and 1 long-term post-acute unit) with patient-sharing. Patients with CRAB in clinical or surveillance cultures from January 2021 to April 2025 were included. Active surveillance was conducted in select units using rectal swabs, and available isolates underwent genomic sequencing. **Results:** We identified 13 unique patients with 61 NDM-CRAB clinical (7 [11%]) or surveillance (54 [89%]) cultures. Two of 10 colonized patients subsequently developed clinical infection. NDM-CRAB constituted 25% of all sequenced CRAB isolates during this time. Among the 13 patients, median age was 62 years, 6 (46%) were female, all had prior healthcare exposure and 10 (77%) had colonization or infection with other multidrug-resistant organisms (MDROs). None reported international travel. Nine patients (69%) had prior exposure to carbapenems, 9 (69%) had central venous catheters, and 7 (54%) required mechanical ventilation. Sites of infection included pneumonia, urinary tract, and wound. Following sporadic cases from 2021-2023, a cluster of 2 cases occurred on the post-acute unit in 2024 followed by a prolonged outbreak (n=10) detected via weekly rectal surveillance cultures (Figure). With the exception of the index case, all other post-acute unit outbreak cases had a prior negative surveillance screen, suggesting transmission among long-stay patients. Observations revealed suboptimal environmental surface-cleaning and deficiencies in use of personal protective equipment. No additional cases were identified on other units including ICUs undergoing routine weekly surveillance. Twenty-five NDM-CRAB isolates underwent genomic sequencing. All belonged to the same A. baumannii multilocus sequencing typing (MLST) lineage (STPas2, STOxf 218/2164). Long-read sequencing of select isolates identified blaNDM-1 and blaOXA-23 integrated in the chromosome. Notably, one isolate additionally acquired blaOXA-72 on a r3-T60 Acinetobacter plasmid, suggesting continuing genetic evolution. **Conclusion:** Over a 5-year period, we found NDM-CRAB transmission in a post-acute care unit within an acute care hospital, among long-stay patients with invasive devices and antibiotic exposure. We did not observe sustained transmission in other parts of the hospital, underscoring the contribution of long-term acute care in MDRO dissemination. Genomic analysis helped distinguish this cluster from other non-NDM CRAB cases, supplementing epidemiologic findings.

**Figure:**